# FGF10 and Lipofibroblasts in Lung Homeostasis and Disease: Insights Gained From the Adipocytes

**DOI:** 10.3389/fcell.2021.645400

**Published:** 2021-05-26

**Authors:** Yu-Qing Lv, Qhaweni Dhlamini, Chengshui Chen, Xiaokun Li, Saverio Bellusci, Jin-San Zhang

**Affiliations:** ^1^Key Laboratory of Interventional Pulmonology of Zhejiang Province, Center for Precision Medicine, The First Affiliated Hospital of Wenzhou Medical University, Wenzhou, China; ^2^International Collaborative Center on Growth Factor Research, School of Pharmaceutical Sciences, Wenzhou Medical University, Wenzhou, China; ^3^Cardio-Pulmonary Institute, Institute of Lung Health and Department of Pulmonary and Critical Care Medicine and Infectious Diseases, Universities of Giessen and Marburg Lung Center (UGMLC), Member of the German Center for Lung Research (DZL), Justus-Liebig University Giessen, Giessen, Germany

**Keywords:** FGF10, adipocytes, adipocyte-like cells, stem cell, lipofibroblast, myofibroblast, lung regeneration/repair

## Abstract

Adipocytes not only function as energy depots but also secrete numerous adipokines that regulate multiple metabolic processes, including lipid homeostasis. Dysregulation of lipid homeostasis, which often leads to adipocyte hypertrophy and/or ectopic lipid deposition in non-adipocyte cells such as muscle and liver, is linked to the development of insulin resistance. Similarly, an altered secretion profile of adipokines or imbalance between calorie intake and energy expenditure is associated with obesity, among other related metabolic disorders. In lungs, lipid-laden adipocyte-like cells known as lipofibroblasts share numerous developmental and functional similarities with adipocytes, and similarly influence alveolar lipid homeostasis by facilitating pulmonary surfactant production. Unsurprisingly, disruption in alveolar lipid homeostasis may propagate several chronic inflammatory disorders of the lung. Given the numerous similarities between the two cell types, dissecting the molecular mechanisms underlying adipocyte development and function will offer valuable insights that may be applied to, at least, some aspects of lipofibroblast biology in normal and diseased lungs. FGF10, a major ligand for FGFR2b, is a multifunctional growth factor that is indispensable for several biological processes, including development of various organs and tissues such as the lung and WAT. Moreover, accumulating evidence strongly implicates FGF10 in several key aspects of adipogenesis as well as lipofibroblast formation and maintenance, and as a potential player in adipocyte metabolism. This review summarizes our current understanding of the role of FGF10 in adipocytes, while attempting to derive insights on the existing literature and extrapolate the knowledge to pulmonary lipofibroblasts.

## Introduction

FGF10, a member of the FGF family, is a potent mitogen that is indispensable for proper development, regeneration, and health. In general, FGFs elicit biological responses by binding to and activating four highly conserved transmembrane tyrosine kinase receptors (FGFR1–4). Currently, the FGF family comprises at least 22 members that are classified into seven subfamilies based on sequence homology, functional properties, and evolutionary phylogeny. FGF10 belongs to the FGF7 subfamily, a group consisting of FGF3, 7, 10, and 22 (Ornitz and Itoh, 2015), and binds with higher affinity to FGFR1b and FGFR2b compared to the other FGF receptors (Bellusci et al., 1997). More details regarding FGF10, including its protein structure and function, expression profile in tissues or during development, and signaling transduction mechanisms are comprehensively reviewed elsewhere (Ndlovu et al., 2018). Among the FGFs, Fgf10 is well-characterized for its roles in development of various organs and tissues, including the lung and white adipose tissue (WAT) (Gartside et al., 2009; Ohta and Itoh, 2014; Ornitz and Itoh, 2015). Mice lacking *Fgf10* or its primary receptor *Fgfr2b* display multiple organ defects, including complete lung agenesis (Min et al., 1998; De Moerlooze et al., 2000; Ohuchi et al., 2000). *FGF10* expression was observed in adipose tissue and has been strongly implicated in adipogenesis (Sakaue et al., 2002; Patel et al., 2005; Ohta and Itoh, 2014). Significantly, *Fgf10* knockout (KO) mice display impaired WAT development, indicating that Fgf10 expression is crucial for normal WAT development (Yamasaki et al., 1999; Sakaue et al., 2002). Herein, we summarize the current knowledge and gaps in our understanding of the role of FGF10 in adipocytes and adipocyte-like cells.

## Succinct Background on Adipose Tissue and Adipogenesis

The adipose tissue (AT) is a dynamic metabolic and endocrine organ that contributes to various crucial physiological processes, including regulation of energy balance and metabolic homeostasis. In mammals, AT is classified into two morphologically and functionally distinct types: WAT and brown adipose tissue (BAT) (Rosen and Spiegelman, 2000). WAT consists generally of spherical cells that contain a large uniocular lipid droplet occupying the majority of the cytosol and markedly lower mitochondria content than brown adipocytes (Cannon and Nedergaard, 2004; Cinti, 2005). WAT primarily stores excess energy in the form of triglycerides and mobilizes the energy depending on the state of energy balance and immediate physiological needs. Furthermore, WAT secretes several endocrine factors, such as adipokines and cytokines, that act on various targets to regulate multiple metabolic processes (Ahima and Lazar, 2008). BAT, in contrast to WAT, consists of ellipsoidal cells that contain multiple, small, multilocular lipid droplets, and higher content of UCP1-expressing mitochondria (Cannon and Nedergaard, 2004; Cinti, 2005). BAT mainly dissipates chemical energy *via* UCP1-mediated mitochondrial uncoupling, generating heat in the process; a phenomenon often dubbed “non-shivering thermogenesis” (Lowell and Flier, 1997; Cannon and Nedergaard, 2004; Richard and Picard, 2011).

**Figure 1:**
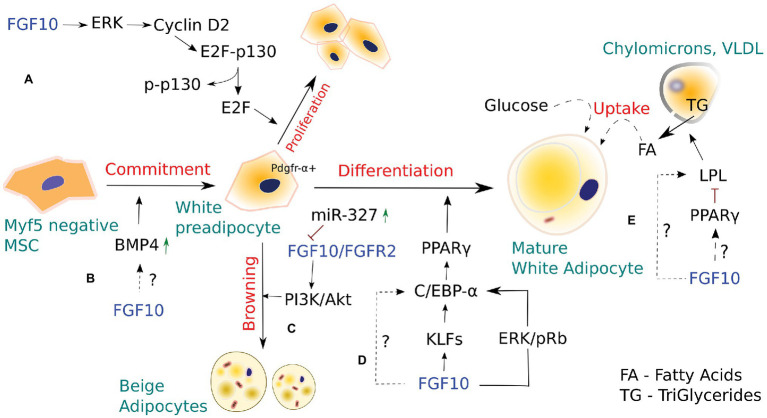
The role of FGF10 in white and Beige adipocyte development. **(A)** FGF10-stimulated activation of the Ras/MAPK pathway induces proliferation of white preadipocytes *via* cyclin D2-dependent phosphorylation of p130. **(B)** Bone morphogenic protein 4 (BMP4), acting downstream of FGF10, is known to regulate FGF10-mediated bud outgrowth during branching morphogenesis of the developing lung, thus contributing to controlling bud size. In adipogenesis, BMP4 stimulates commitment of pluripotent stem cells toward the white adipocyte lineage. However, whether a dynamic interplay between FGF10 and BMP4 occurs in adipogenesis, similar to that seen in embryonic lung development, remains to be elucidated. **(C)** FGF10 is part of a miRNA-327-regulated autocrine loop that stimulates development of Beige adipocytes from Pdgfrα+ white preadipocytes. However, Beige adipocytes are also thought to arise from mature white adipocytes *via* β-3 adrenergic stimulation, cold exposure, and chronic PPAR-γ induction. **(D)** FGF10, *via* a number of signaling routes, activates downstream PPAR-γ, which in turn induces white adipocyte differentiation. **(E)** FGF10 overexpression in intramuscular adipocytes enhanced the expression of lipoprotein lipase (LPL) and LPL-induced lipid accumulation, suggesting a possible role for FGF10 in regulating adipocyte metabolism. However, LPL is also known to be regulated by PPAR-γ, and thus whether FGF10 would target LPL directly or *via* PPAR-γ to exert its effects in adipocyte metabolism remains to be determined.

Adipogenesis is a multi-step process by which mature adipocytes arise from mesenchymal stem cells (MSCs) through cell differentiation. This process is regulated by a complex network of transcription factors in concert with several extracellular mediators, such as hormones and growth factors, and occurs in two major phases. First, MSCs commit to preadipocytes, and second, preadipocytes terminally differentiate into mature, functional adipocytes (Rosen and Macdougald, 2006). Adipocytes represent the primary building blocks of adipose tissue, and three types of adipocytes deriving from distinct MSC lineages constitute two main types of adipose tissue. White adipocytes mainly derive from the Myf5-negative lineage, while brown adipocytes arise from the Myf5-positive lineage. Some studies, however, indicate that Myf5-positive precursors may give rise to a subset of white adipocytes as well (Sanchez-Gurmaches and Guertin, 2014; Sebo et al., 2018). In addition to classical brown adipocytes, a distinct type of thermogenic, brown adipocyte-like cells known as Beige adipocytes have been found in WAT depots (Cannon and Nedergaard, 2004; Wu et al., 2012). Beige adipocytes are thought to arise from: (a) mature white adipocytes *via* β3 adrenergic stimulation, chronic Peroxisome proliferator-activated receptor gamma (PPAR-γ) induction, and cold exposure-induced trans-differentiation (Barbatelli et al., 2010), (b) platelet-derived growth factor receptor α (PDGFRα) positive white preadipocytes *via* differentiation (Figure 1; Fischer et al., 2017), and (c) Myf5 negative MSCs (Sanchez-Gurmaches et al., 2016). Beige and brown adipocytes share several biochemical characteristics, specifically, they both contain abundant UCP1-expressing mitochondria and multilocular lipid droplets.

## Role of FGF10 in Adipogenesis and WAT Browning

Autocrine/paracrine FGF10 signaling has been implicated in WAT development, remodeling, and metabolism (Ohta and Itoh, 2014). FGF10 is shown to play a crucial role in the differentiation of preadipocytes in WAT *via* activation of *PPAR-γ*, the key transcriptional regulator of adipogenesis (Sakaue et al., 2002; Asaki et al., 2004). KO mice studies revealed that loss of Fgf10 expression in embryonic WAT results in markedly reduced expression of *PPAR-γ*, indicating that FGF10 acts upstream of *PPAR-γ* to stimulate adipogenesis (Ohta et al., 2011). Furthermore, blockade of Fgf10 signaling inhibited the expression of C/EBPα, a major adipogenic transcription factor that is critical for the initiation of 3T3-L1 cell differentiation, thereby suppressing adipocyte development in differentiating 3T3-L1 preadipocytes (Lane et al., 1999; Sakaue et al., 2002). These data suggest that FGF10 expression or activity is critical for preadipocyte differentiation into mature adipocytes. Although FGF10 is shown to promote adipogenesis/terminal adipocyte differentiation *via* upregulation of the adipogenic genes *PPAR-γ*, C/EBPα, and lipoprotein lipase (LPL), it remains unclear whether FGF10 may induce/regulate adipogenic cell fate determination in undifferentiated MSCs, as most, if not all studies investigating the role of Fgf10 in adipogenesis have employed the 3 T3-L1 cell line, which is already committed to adipocyte lineage. Thus, further studies employing multipotent undifferentiated MSC cell lines, are warranted to investigate the potential effects of FGF10 on adipogenic cell fate commitment of MSCs *in vivo*.

FGF10 is abundantly expressed in the Stromal Vascular Fraction of WAT, which is comprised of adipocyte stem and progenitors among other cell types, suggesting that FGF10 is essential for adipogenesis *in vivo* (Yamasaki et al., 1999). Classical observations indicate that FGF10 expression in WAT stimulates both proliferation and differentiation of preadipocytes *via* the Ras/MAPK pathway (Figure 1; Asaki et al., 2004; Konishi et al., 2006; Ohta et al., 2011). In preadipocytes, FGF10/FGFR2b signaling *via* the Ras/MAPK cascade was shown to trigger pRb and subsequent Rb-C/EBP complex formation, leading to downstream activation of *PPAR-γ*, which in turn induces adipogenesis. FGF10 was also shown to mediate preadipocyte proliferation in WAT by inducing cyclin D2-dependent phosphorylation of P130 through the Ras/MAPK pathway. In support of these observations, *Fgf10* KO embryos exhibited aberrant *Cyclin D2* expression and P130 phosphorylation in the WAT and markedly impaired preadipocyte proliferation (Konishi et al., 2006; Ohta et al., 2011).

In addition, specific blockade of Fgf10/Fgfr2 and downstream AKT signaling with miR-327, a key regulator of preadipocyte differentiation into Beige adipocytes, resulted in impaired preadipocyte differentiation, indicating that Fgf10 may indeed drive preadipocyte differentiation *via* the Fgfr2-Akt axis. Furthermore, *miR-327* was shown to regulate *Fgf10*-mediated preadipocyte differentiation by inhibiting the Fgf10-activated Fgfr2-Akt signaling cascade (Fischer et al., 2017). However, using the established mouse 3T3-L1 cell line to investigate the effect of *Fgf10* and *miR-327* levels on preadipocyte proliferation, the researchers demonstrated that Fgf10 expression levels do not affect preadipocyte proliferation (Fischer et al., 2017).

Fischer et al. (2017) reported the existence of a *miRNA-327*-FGF10-FGFR2 autocrine regulatory loop in PDGFRα+ cells, which is critical for WAT browning. They demonstrated, for the first time, that a mechanism involving FGF10 could regulate PDGFRα+ preadipocyte differentiation into thermogenic Beige adipocytes. These findings are particularly intriguing because PDGFRα+ preadipocytes, similar to mesenchymal progenitors of pulmonary lipofibroblasts (LIFs), not only differentiate *via* a mechanism involving Fgf10 upregulation but also express a number of shared adipogenic genes. Considering the similarities between beige and LIF progenitors, we postulate that, similar to beige/white adipocyte‐ and bipotent PDGFRα+ progenitors, LIF progenitors, *via* Fgf10 upregulation, may also give rise to distinct LIF cell subtypes that may play some as yet undiscovered roles in lung biology.

## Other FGF Family Members are Implicated in Adipose Tissue Development, Remodeling, and Function

Apart from FGF10, other FGF family members, including FGF21 and FGF9, play crucial roles as autocrine/paracrine adipokines in regulating energy homeostasis by influencing WAT or BAT/beige development, remodeling, and function. FGF21 has been shown to activate thermogenesis *via* induction of WAT browning and thermogenic genes, such as PGC1 and UCP1, following cold exposure (Fisher et al., 2012). FGF21 KO mice exhibited an impaired adaption to chronic cold exposure and diminished WA browning, suggesting that FGF21 is critical for normal adaptations to cold exposure. In addition, FGF21 *via* a feed-forward loop involving *PPAR-γ*, regulates insulin sensitivity and glucose homeostasis in WAT (Dutchak et al., 2012), suggesting that FGF21 contributes to the antidiabetic activities of *PPAR-γ*. On the contrary, FGF9 upregulation was shown to negatively regulate WAT browning and the expression of thermogenic genes, including PGC1and UCP1, and positively correlate with obesity (Sun et al., 2019). The inhibitory effects of FGF9 on WAT browning were accompanied with decreased expression of adipogenic markers including *C/EBPβ* and *PPAR-γ*, indicating that FGF9 upregulation inhibits adipogenesis. Besides FGF21 and FGF9, other FGFs such as FGF1, FGF16, and FGF19, also have important roles in adipogenesis; however, due to space limitations, these growth factors will not be covered here. Taken together, these findings provide new insights for FGFs as potential targets for treating obesity and its related metabolic disorders.

## FGF10 in the Regulation of Multiple Adipocyte Metabolic Factors

### Lipoprotein Lipase

Lipoprotein lipase, a member of the lipase superfamily, is chiefly expressed in tissues that oxidize or store large amounts of fatty acids, such as WAT and BAT, and is an important early marker of adipocyte differentiation (Amri et al., 1996). LPL hydrolyzes triglycerides from circulating triglyceride-rich lipoproteins (VLDL and chylomicrons) into fatty acids, which are then taken up by adipocytes for storage or energy production *via* beta-oxidation (Mead et al., 2002). Specific siRNA-mediated knockdown of *Fgf10* in goat intramuscular preadipocytes inhibited lipid droplet accumulation, and impaired adipocyte development, suggesting that Fgf10 plays a pivotal role in adipocyte development and metabolism, as lipid accumulation represents a major functional characteristic of adipocytes (Xu et al., 2018). Beyond its adipogenesis activity, *PPAR-γ* is involved in various aspects of adipocyte lipid metabolism and regulates the expression of adipocyte-specific genes associated with lipid accumulation and metabolism, including LPL (Schoonjans et al., 1996; Kersten et al., 2000). Interestingly, Fgf10 overexpression in intramuscular adipocytes enhanced the expression of LPL among other transcription factors, while *Fgf10* knockdown using RNA interference suppressed the expression of LPL and LPL-induced lipid accumulation (Xu et al., 2018). These findings suggest a possible role for FGF10 in regulating adipocyte metabolism while raising the question of whether FGF10 would target LPL directly or *via PPAR-γ* to exert its effects in adipocyte metabolism.

### Kruppel-Like Factors

Kruppel-like factors (KLFs) are a large family of zinc-finger proteins with functionally diverse roles in various physiological and cellular processes, including adipogenesis (Pearson et al., 2008; Pei et al., 2011). KLFs constitute the central transcriptional cascade that acts sequentially and upstream of the adipogenic master regulator *PPAR-γ* to negatively or positively regulate adipogenesis (Wu and Wang, 2013). Recently, *Fgf10* was shown to upregulate mRNA expression of KLF3, 9, and 13 in goat intramuscular preadipocytes, indicating a possibility that *Fgf10* may regulate adipocyte development, at least in part, by targeting KLF3, 9, and 13 (Xu et al., 2018). However, further studies are needed to clarify the precise effects of these KLFs on adipogenesis, and to establish the specific mechanisms involved (Xu et al., 2018).

### Bone Morphogenic Proteins

Bone morphogenic proteins (BMPs) are known to play crucial roles in several hallmarks of adipogenesis, including adipocyte lineage commitment, preadipocyte differentiation, and support of adipocyte function (Fux et al., 2004; Huang et al., 2009; Schulz et al., 2011). BMPs are polypeptide growth factors that belong to the TGF-β superfamily and are involved in several aspects of embryonic lung patterning and adipogenesis. Among the BMP family members, BMP2, BMP4, and BMP7 are the most studied factors for their roles in adipogenesis. BMP2 and BMP4 are essential for white adipocyte development (Fux et al., 2004; Huang et al., 2009), while BMP7 is involved in brown adipocyte development (Schulz et al., 2011). BMP4 plays a critical role in adipogenesis and has been shown not only to stimulate commitment of pluripotent stem cells toward the white adipocyte lineage but also promote WAT differentiation (Otto et al., 2007). Interestingly, BMP4 was identified to act in concert with FGF10 during lung development. BMP4, acting downstream of FGF10, regulates branching and patterning of the developing lung, thus contributing to bud size control. During branching morphogenesis, FGF10 induces bud formation in proximal-distal regions and subsequent epithelial expression of BMP4, which in turn limits further FGF10-mediated bud outgrowth (Weaver et al., 2000). Given that both FGF10 and BMP4 have been implicated in adipogenesis, it will be interesting to study whether a dynamic interplay between FGF10 and BMP4 occurs in adipogenesis, for instance, as similar to that seen in embryonic lung development.

## The Roles of FGF10 in Adipocytes May be Extrapolated to Lipofibroblasts

Lipofibroblasts and adipocytes share several common characteristics, despite their distinct anatomical locations. For one thing, both LIFs and adipocytes are lipid-laden cells that characteristically express an array of adipogenic genes and markers, such as Adrp, *PPAR-γ*, and LPL, associated with various aspects of their biological processes, including lipid metabolism and lipogenesis. The roles of some of these markers in LIF and adipocyte cellular biology are discussed in detail in other sections of this review. Furthermore, LIFs and adipocytes utilize shared developmental signaling pathways during their formation, including Fgf10, *PPAR-γ*, and PDGFRα signaling. Both cell types emerge from MSCs *via* Fgf10-mediated differentiation of their mesenchymal progenitors into mature cells (Ohta et al., 2011; Al Alam et al., 2015). In addition, like lipofibroblasts, adipocytes require *PPAR-γ* signaling during their maturation and for maintenance of their phenotype. Moreover, apart from FGF10 and *PPAR-γ*, the mesenchymal progenitors of both LIFs and adipocytes express PDGFRα, whose expression levels progressively decrease during their course of differentiation.

Given the many similarities between adipocytes and LIFs, it is plausible to draw a comparison between the two cells. We, therefore, postulate that the established and potential roles of FGF10 signaling in adipocytes can be extrapolated to pulmonary LIFs. While earlier studies have also predicted the role of Fgf10 in LIF formation by extrapolating from its role in adipogenesis (Al Alam et al., 2015), further studies to test whether, with regard to FGF10 signaling, the similarities between LIFs and adipocytes extend from cellular development to other aspects of their biology. Such studies may help unravel more mysteries of LIF biology and function in lung development, homeostasis, and regeneration.

During embryonic lung development, LIFs originate from several progenitor pools, including a subset of FGF10-expressing cells in the lung mesenchyme (Al Alam et al., 2015). Lineage tracing of Fgf10-positive (FGF10+) cells using *Fgf10* knock-in mice revealed that mesenchymal-derived FGF10+ progenitor cells give rise to multiple lineages, including myogenic and adipogenic lineages (El Agha et al., 2014). Furthermore, FGF10 signaling was shown to promote the adipogenic differentiation potential of MSCs through preferential induction of LIF (adipogenic) differentiation rather than alveolar MYF (myogenic) differentiation during lung development (El Agha et al., 2014), suggesting that FGF10 signaling may control early cell fate decisions of MSCs as well as induce conversions between established lineages. The possibility that FGF10 signaling may regulate MSC fate would have profound implications, especially in the context of lung repair and regeneration following injury or disease.

## FGF10-FGFR2B Signaling in Lipofibroblasts – Alveolar Type 2 Cells Two-Way Communication

Pulmonary lipofibroblasts were first described five decades ago, and since this initial landmark report, various research groups have invested their efforts to characterize their development, morphology, and their biological roles *in vivo*. The capacity of LIFs to recruit, store, and supply alveolar type 2 (AT2) cells with lipid substrates for lung surfactant production (Rehan and Torday, 2014), has offered exciting opportunities to make important discoveries that will impact human health.

*In vivo* tracer studies have shown that LIF cells usually reside next to AT2 cells. Therefore, LIF cells are considered to be essential components of AT2 stem cell niches, and an essential factor in maintaining AT2 cell stemness (Barkauskas et al., 2013; El Agha and Bellusci, 2014; Al Alam et al., 2015). A myriad of studies indicate that AT2 cells and juxtaposed LIFs interact reciprocally *via* paracrine signaling pathways, such as the PPAR-γ pathway, in coordinating normal development, homeostasis, and regeneration/repair of the distal lung (Tordet et al., 1981; El Agha et al., 2014).

Apart from LIFs, FGF10 has been implicated in promoting AT2 lineage formation. Chao et al. (2017) found that lungs of *Fgf10*^+/−^ mice had decreased total number of epithelial cells compared to wild-type mice, and unbalanced alveolar epithelial cell population, with a decreased proportion of AT2 cells and an increased proportion of AT1 cells. Furthermore, FGF10 hypomorphic mice with significantly reduced FGF10 expression levels exhibited marked AT2 defects compared to normal wild-type mice (Chao et al., 2017), further indicating that FGF10 plays a vital role in AT2 lineage formation.

AT2 cells normally play essential roles in host defense, barrier function, and normal lung homeostasis/repair. AT2 cells are generally small, rounded, or cuboidal cells containing characteristic lamellar bodies and apical microvilli (Mason, 2006), and distributed between alveolar epithelial type 1 cells (AT1) in the alveolar septa. In normal adult lungs, AT2 cells are more abundant than AT1 cells, accounting for 14–16% of all alveolar epithelial cells, and serve as an important progenitor during repair after lung injury and during normal lung homeostasis. AT1 cells appear to be more susceptible to injury from either endogenous or exogenous factors, while AT2 cells are more resistant. In severe cases of lung injury, such as the COVID-19 infection (Chen et al., 2020), the alveolar epithelium usually exhibits widespread AT1 cell necrosis, characterized by a denuded alveolar basement membrane and formation of hyaline membranes, one of the hallmark features of acute respiratory distress syndrome (ARDS).

**Figure 2:**
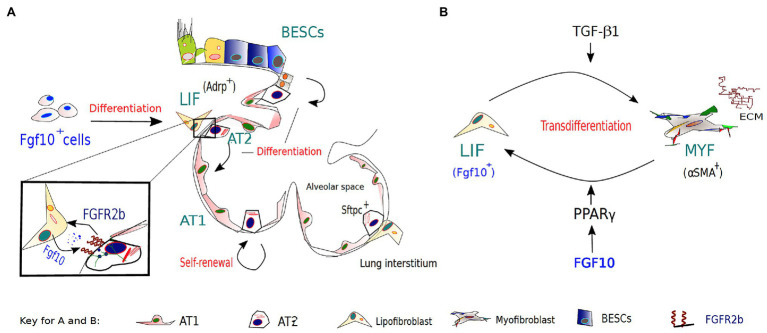
The role of FGF10-FGFR2B signaling in lipofibroblast (LIF)-AT2 mediated alveolar repair and fibrosis development/resolution. **(A)**. During embryonic lung development, a subset of FGF10-expressing cells in the lung mesenchyme gives rise to LIFs, which are considered to be essential components of AT2 stem cell niches. AT2 cells and neighboring LIFs interact reciprocally *via* paracrine signaling pathways, such as the PPAR-γ pathway, in coordinating normal development, homeostasis, and regeneration/repair of the distal lung. Apart from other reserve stem cell populations for the alveolar epithelium, AT2 cells are important progenitors during repair after lung injury and during normal lung homeostasis. **(B)** The origins of activated myofibroblasts (MYF) in fibrotic lungs remain speculative; however, FGF10-expressing LIFs have been implicated as a novel source of the activated myofibroblasts. Following bleomycin-induced injury, lipofibroblasts are thought to transdifferentiate into activated myofibroblasts, from which a subpopulation reverts to a quiescent phenotype characteristic of the pre-existing LIFs during fibrosis resolution.

Following epithelial injury resulting in loss of both AT1 and AT2 cells, surviving AT2 migrate and undergo compensatory proliferation along the alveolar septa, in an attempt to repopulate the denuded epithelial barrier, and eventually transdifferentiate to replace lost AT1 cells (Kim et al., 2006). Recent findings, however, suggest that although AT2 cells can contribute to alveolar epithelial regeneration after injury, some of the newly generated alveolar epithelial cells (AECs) may arise from activated Bronchial epithelial stem cells (BESCs; Yuan et al., 2019). In a bleomycin model of lung injury, BESCs function as a source of alveolar epithelial regeneration and repair by differentiating toward AT1 and AT2 cell lineages over of Basal cells (BCs) in honeycomb cysts, in response to increased FGF10 signaling (Yuan et al., 2019). More on these findings will be further discussed in the FGF10 and respiratory disease section. Overall, these observations suggest that elevated FGF10 levels in BESCs may induce efficient epithelial repair *via* BESCs surrogacy for incapacitated AT2 progenitors in case of severe lung injury (Figure 2A) and that FGF10-based therapies based on this phenomenon may prove beneficial for ARDS and/or COVID-19 patients.

## FGF10 in the LIF to MYF Switch During Fibrosis Formation and Resolution

Myofibroblasts (MYF) play an important role in both physiological and pathological repair processes (Kis et al., 2011; Hinz et al., 2012). In physiological repair, myofibroblasts are transiently activated to facilitate wound closure by producing extra cellular matrix (ECM) proteins, notably fibronectin and collagens type I and III (Tomasek et al., 2002; Klingberg et al., 2013). Successful wound repair is followed by degradation of the provisional matrix and disappearance of myofibroblasts *via* a number of mechanisms, including apoptosis. In pathological repair, however, myofibroblasts are continuously activated due to ongoing injury, often leading to excessive deposition of aberrant ECM and abnormal tissue repair. Moreover, these persistent myofibroblasts tend to resist apoptosis and, hence, their physiological clearance. Myofibroblast persistence leads to pathological scar formation, and ultimately organ fibrosis.

In pulmonary fibrosis, the origins of activated myofibroblasts remain speculative; however, several sources have been implicated. Recently, lineage-tracing studies implicated FGF10-expressing LIFs as a novel source of the activated MYFs (El Agha et al., 2017b). These studies not only indicated that LIFs transdifferentiate into activated MYFs following bleomycin-induced injury but also that a subpopulation of the myofibroblasts reverts to a quiescent phenotype characteristic of the pre-existing LIFs during fibrosis resolution. Unraveling the origins and/or fate of activated myofibroblasts during fibrosis resolution offers the unique opportunity to clearly define pathological hallmarks of lung fibrosis, and discover novel therapeutic interventions that may inhibit or even reverse the switch between lipogenic and myogenic phenotype, and ultimately accelerate fibrosis regression.

Furthermore, activation of PPAR-γ signaling *via* rosiglitazone (a potent PPAR-γ agonist) treatment antagonized TGF-β-mediated myofibroblast differentiation likely by reinforcing the lipogenic phenotype (El Agha et al., 2017b). Interestingly, TGF-β upregulation has been found to be induced by SARS-Cov-2 infection (Agrati et al., 2020; Chen, 2020; Ferreira-Gomes et al., 2021) and associated with several complications of severe COVID19, such as ARDS and pulmonary fibrosis. Thus, it is plausible that FGF10, likely *via PPAR-γ* activation, gets involved in an attempt to replenish LIFs lost to unrestrained MYF differentiation – driven by TGF-β upregulation and chronic epithelial injury (Figure 2B). The possibility that FGF10, *via PPAR-γ* signaling, can drive reinforcement of the lipogenic phenotype at the expense of the myogenic phenotype raises the potential that FGF10-based therapies could be beneficial in combating idiopathic pulmonary fibrosis (IPF) and/or COVID-19 related pulmonary fibrosis.

## FGF10-FGFR2B Signaling and Other Associated Respiratory Diseases

Aberrant FGF10-FGFR2B signaling contributes to the pathophysiology of multiple respiratory diseases, including IPF and bronchopulmonary dysplasia (BPD). IPF is a chronic age-related respiratory disease of unknown etiology belonging to a heterogeneous group of interstitial lung diseases (ILDs) that are characterized by distorted pulmonary architecture, compromised lung function, and respiratory failure. By way of background, it is worth emphasizing that IPF differs from COVID-19 related pulmonary fibrosis in regard to certain etiological aspects, despite both being characterized by aberrant accumulation of myofibroblasts, reduced lung compliance, and excessive deposition of collagen and other ECM (Sime and O’Reilly, 2001; King et al., 2011; Steele and Schwartz, 2013). More importantly, pulmonary fibrosis is a common consequent manifestation of several acute and chronic ILDs such as ARDS – a major cause of morbidity and mortality in COVID-19 patients (Kruglikov and Scherer, 2020).

Interestingly, apart from fibrotic lungs, reduced lung compliance, as well as AT2 cells with altered surfactant protein expression, have been observed in different models of obesity (Inselman et al., 2004; Foster et al., 2010; Correll et al., 2019). The association of obesity with lung pathologies is gaining much attention, as obesity is increasingly implicated as a major risk factor in several lung disorders, including pulmonary fibrosis and severe COVID-19 infection. In an obese state, SARS-Cov-2 infection is thought to additionally modify the already-compromised adipocytes and adipocyte-like cells by altering their lipid metabolism and promoting their adoption of a differentiation-prone cell state, thus negatively affecting global metabolic homeostasis. In severe cases of SARS-Cov-2 infection, this may involve the lipogenic-to-myogenic switch, a key event in pulmonary fibrogenesis (Kruglikov and Scherer, 2020). Moreover, obese IPF patients have significantly higher waitlist mortality and post-transplant mortality of just over 3 months, compared with controls (Nathan et al., 2010; Gries et al., 2015). In addition, obesity is associated with worse outcome of COVID-19 infection, dysregulated lipid metabolism, and or ectopic lipid accumulation in non-adipocytes, such as pulmonary LIF and neighboring AT2 cells. Therefore, understanding LIF‐ and or AT2-cell behavior in the context of obesity and COVID-19 may unlock the mysteries associated with COVID-19 pathogenesis.

Dysregulated lipid metabolism in AT2 cells has been linked to an altered surfactant profile (Schmidt et al., 2002; Foster et al., 2010), which, we hypothesize, would increase AT2 cell susceptibility to COVID-19 infection, as surfactant proteins are known to participate in host defense against viral infection (Hsieh et al., 2018). Similarly, ectopic accumulation of lipid in AT2 cells has been shown to induce AT2 hyperplasia (Foster et al., 2010), which, we again hypothesize, may translate to more ACE2-expressing AT2 cells – the primary target of the COVID-19 virus (Zhao et al., 2020); that is, increased number of viral entry points, and thus, increased susceptibility of AT2 cells to COVID-19 infection. Furthermore, given the reciprocal interactions between LIFs and neighboring AT2 cells, in which LIFs traffic lipid substrates for surfactant production to AT2 cells (Rehan and Torday, 2014), it is highly plausible that aberrant lipid accumulation in AT2 cells and subsequent AT2 hyperplasia may also stem from impaired lipid trafficking from LIFs to AT2 cells.

In addition, obesity-associated AT2 hyperplasia may also explain the histologic features of samples derived from IPF lungs, as well as patients with severe COVID-19 cases (Mora et al., 2005; Selman and Pardo, 2006; Wigén et al., 2020), showing distinct foci of hyperplastic AT2 cells. Moreover, in obese lungs, LIFs, and AT2 cells alike, extensively accumulate lipid droplets (Foster et al., 2010), which in turn may lead to lipotoxicity, and lipotoxicity-mediated outcomes, such as cell cycle arrest and apoptosis, often observed in IPF among other lung pathologies.

FGF10-FGFR2b signaling has been shown to promote the resolution of pulmonary fibrosis *via* a number of mechanisms, including the one we mentioned earlier in this review (El Agha et al., 2017b). Although the precise role of FGF10 in IPF pathology remains unclear, studies found significantly elevated FGF10 expression levels in lung tissues of IPF patients compared to non-IPF donors. Strikingly, fibrotic foci, in contrast to fibrotic lesions, had detectable FGF10 protein expression, but rather at lower levels (El Agha et al., 2017a). Based on these observations, some have speculated that FGF10 is unlikely to be involved in the initial triggering events of IPF, but rather inducted to promote fibrosis resolution by counteracting the effects of TGF-β1 (Gupte et al., 2009; El Agha et al., 2017b).

In a more recent study using bleomycin injury models, FGF10 overexpression in BESCs was shown to enhance fibrosis resolution by promoting alveolar epithelial regeneration over the development of fibrotic honeycomb lesions, while inactivation of its receptor FGFR2b led to impaired epithelial regeneration by BESCs (Yuan et al., 2019). The need for elevated FGF10 levels to induce BESCs-driven AEC regeneration and fibrosis resolution following lung injury may explain why IPF is tightly correlated with aging, as FGF10 expression is known to decrease with age. Taken together, investigating the specific role of FGF10 and its receptor FGFR2B in IPF may help create effective anti-fibrotic FGF10-based therapies that precisely target this signaling pathway with optimal efficacy.

Deficient FGF10 signaling during embryonic lung development is associated with BPD, a lethal lung developmental disorder of prematurely born infants. Hyperoxia injury models of BPD in *Fgf10* heterozygous pups resulted in complete postnatal lethality, starting at day 5, while in normoxia, no postnatal lethality was observed (Chao et al., 2017). This suggests that a potential beneficial effect of recombinant FGF10 treatment is likely to be effective in the context of BPD. This could, in turn, translate into the development of novel FGF10-based therapies that may improve the prognosis of BPD in preterm neonates.

## Conclusion and Perspective

By looking at the numerous developmental and functional similarities between pulmonary LIFs and adipocytes, especially white adipocytes, a few lessons have emerged. We believe that these lessons, particularly the findings pertaining to adipocyte biology, including our knowledge about FGF10 signaling in these cells, can be extrapolated to explain, at least some aspects of LIF biology in normal and diseased lungs. To achieve this, further studies using scRNA-seq and lineage tracing tools are required to further explore the influence of FGF10 signaling in LIFs, examine the role of LIF progenitors beyond early developmental stages, and better assess the functional heterogeneity of pulmonary LIFs. For instance, the newly generated Fgf10-CreERT2 line that allows lineage tracing of Fgf10-pos cells during development and postnatally may be helpful. Such studies will contribute significantly to our understanding of the role of LIFs in lung health and disease. Also, given the importance of adipocytes and adipocyte-like cells in multiple physiological processes such as regulation of systemic energy homeostasis, and their implications for metabolic diseases such as obesity, a detailed understanding of the mechanisms by which FGF10 functions in these cells may provide profound insights into disease pathophysiology, and lead to development of novel FGF10-based therapies.

## Author Contributions

J-SZ and SB conceived the study and edited and revised the manuscript. Y-QL and QD drafted the manuscript and designed the figures. CC and XL provided the valuable intellectual input. All authors contributed to the article and approved the submitted version.

### Conflict of Interest

The authors declare that the research was conducted in the absence of any commercial or financial relationships that could be construed as a potential conflict of interest.
